# COVID‐19–Induced Acute Pancreatitis: Clinical Profiles, Outcomes, and Prognostic Indicators From a Global Review‐Based Synthesis

**DOI:** 10.1002/hsr2.71417

**Published:** 2025-10-28

**Authors:** Yu‐Jhou Chen, Tsung‐Hsing Chen, Chen‐June Seak, Chang‐Mu Sung, Shu‐Wei Huang, Sen‐Yung Hsieh, Hao‐Tsai Cheng

**Affiliations:** ^1^ Department of Gastroenterology & Hepatology Chang Gung Memorial Hospital, Linkou Chang Gung Medical Foundation Taoyuan City Taiwan (R.O.C.); ^2^ College of Medicine Chang Gung University Taoyuan City Taiwan (R.O.C.); ^3^ Department of Emergency Medicine New Taipei Municipal TuCheng Hospital (Built and Operated by Chang Gung Medical Foundation) New Taipei City Taiwan (R.O.C.); ^4^ Department of Emergency Medicine Chang Gung Memorial Hospital, Linkou Chang Gung Medical Foundation Taoyuan City Taiwan (R.O.C.); ^5^ Division of Gastroenterology and Hepatology, Department of Internal Medicine New Taipei Municipal TuCheng Hospital (Built and Operated by Chang Gung Medical Foundation) New Taipei City Taiwan (R.O.C.); ^6^ Graduate Institute of Clinical Medicine, College of Medicine Chang Gung University Taoyuan City Taiwan (R.O.C.)

**Keywords:** Acute pancreatitis, clinical course, COVID‐19, image, laboratory, outcomes, real‐world data, review, SARS‐CoV‐2, symptoms

## Abstract

**Background and Aims:**

During the coronavirus disease 2019 (COVID‐19) pandemic, acute pancreatitis (AP) in patients with COVID‐19 has attracted considerable attention. While many reports have described “concurrent COVID‐19 and AP” without excluding established etiologies, the concept of “COVID‐19–induced AP” remains less clearly defined. This study aimed to investigate the associations between initial clinical presentations and outcomes in COVID‐19–induced AP, offering real‐world evidence to identify predictors of short‐term prognosis.

**Methods:**

This is a review‐based synthesis for human studies, with literature search (October 2019–March 2022) using PubMed dual strategies, “Medical Subject Headings (MeSH)” and “title/abstract.” COVID‐19–induced AP was defined by the exclusion of other established etiologies for pancreatitis.

**Results:**

We identified 111 patients (median age 39 years) with 11 deaths. Gastrointestinal symptoms preceded admission in 30.6% of cases, while respiratory symptoms preceded admission in 52.3%. Pancreatitis symptoms before admission were associated with younger age, less lymphocyte counts, lower neutrophil‐to‐lymphocyte ratio, and higher radiologic severity (Balthazar score). Initial white blood cell (WBC) count ≥ 14,000/µL and pancreatic necrosis correlated with surgical intervention. Kaplan–Meier analysis demonstrated that overall survival was not influenced by the sequence of gastrointestinal and respiratory symptom onset (concurrent vs. successive; *p* = 0.543) or by whether pancreatitis symptoms developed before or after hospital admission (*p* = 0.228). Multivariate Cox regression identified elevated WBC count (hazard ratio [HR]: 1.013; 95% confidence interval [CI]: 1.000–1.025; *p* = 0.042), AST‐to‐ALT ratio ≥ 2 (HR: 11.052; 95% CI: 1.441–84.770; *p* = 0.021), and surgical intervention (HR: 6.604; 95% CI: 1.581–27.593; *p* = 0.010) as independent mortality predictors.

**Conclusion:**

Unlike “concurrent COVID‐19 and AP (without excluding established AP etiologies),” “COVID‐19–induced AP” showed no survival disparity by symptom chronology. Mortality was linked to leukocytosis, AST‐to‐ALT ratio, and surgical intervention. Standardized terminology distinguishing “COVID‐19–induced AP” from “concurrent COVID‐19 and AP” is essential to ensure comparability of global clinical data and reduce interpretive bias.

## 1 Introduction

Acute pancreatitis (AP) is associated with considerable morbidity, complications, and mortality. Although the overall risk of death is approximately 1% [1], mortality rises dramatically to 30%–40% in the presence of organ failure or pancreatic necrosis [2], leading to substantial healthcare burdens [3, 4]. This condition results from damage to pancreatic acinar cells as well as local and systemic inflammation. Its pathogenesis is considered multifactorial, involving premature trypsinogen activation, calcium overload, endoplasmic reticulum stress, mitochondrial dysfunction, impaired autophagy, and exosome‐mediated pathways [5, 6, 7]. While geographic and cultural differences exist, the predominant etiologies include biliary or pancreatic ductal obstruction and alcohol consumption, with additional causes such as hypertriglyceridemia, iatrogenic injury, hypercalcemia, infection, and autoimmune disease [8, 9].

During the coronavirus disease 2019 (COVID‐19) pandemic, global medical efforts largely focused on respiratory manifestations of severe acute respiratory syndrome coronavirus 2 (SARS‐CoV‐2). Nonetheless, increasing number of reports have documented cases of hyperamylasemia and/or hyperlipasemia (elevated serum amylase and/or lipase levels) as well as idiopathic AP worldwide [10, 11, 12]. Advances in understanding the pathogenesis of SARS‐CoV‐2, particularly the roles of the viral spike (S) protein and its interaction with angiotensin‐converting enzyme 2 (ACE2), have highlighted the systemic involvement and diverse clinical manifestations [13, 14]. Among these, gastrointestinal presentations—including pancreatic injury, likely related to ACE2 expression in pancreatic cells—have been increasingly recognized and widely discussed [15, 16, 17]. Although several cohort studies have examined the incidence, severity, and mortality of concurrent COVID‐19 and AP [18, 19, 20], the relationship between symptom onset, detailed clinical course (including laboratory and imaging findings), and outcomes remains unclear. Moreover, evidence derived from “concurrent COVID‐19 and AP,” without exclusion of other common pancreatitis etiologies, may not accurately represent patients with true “COVID‐19–induced AP.” This study aimed to evaluate the associations between initial clinical presentations and outcomes in COVID‐19–induced AP, providing real‐world evidence to identify predictors of short‐term prognosis.

## 2 Methods

### 2.1 Article Enrollment Diagram

**Figure 1:**
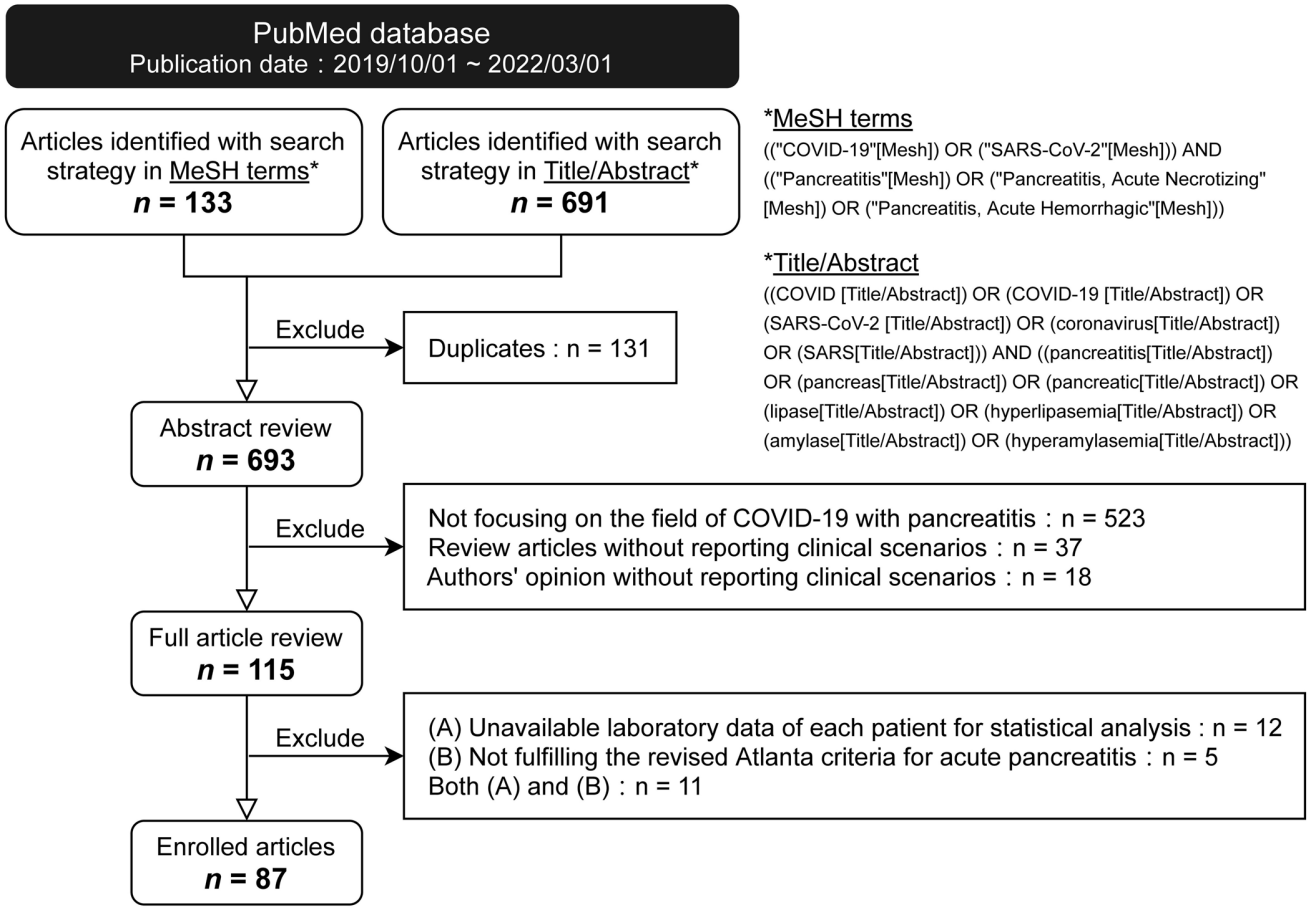
Study enrollment flow diagram. Literature was identified through PubMed searches using Medical Subject Headings (MeSH) terms and title/abstract keywords. Abbreviations: COVID‐19, coronavirus disease 2019; MeSH, Medical subject headings; SARS‐CoV‐2, severe acute respiratory syndrome coronavirus 2.

This study is a review‐based synthesis. Two investigators conducted independent searches of the PubMed database using two strategies: Medical Subject Headings (MeSH) terms and title/abstract keywords (Figure 1). The search encompassed human studies published between October 1, 2019, and March 1, 2022, without language restrictions. For the MeSH strategy, the following terms were applied: *((“COVID‐19”[Mesh]) OR (“SARS‐CoV‐2”[Mesh])) AND ((“Pancreatitis”[Mesh]) OR (“Pancreatitis, Acute Necrotizing”[Mesh]) OR (“Pancreatitis, Acute Hemorrhagic”[Mesh]))*. For the title/abstract search, the following keywords were used: *((COVID[title/abstract]) OR (COVID‐19[title/abstract]) OR (SARS‐CoV‐2[title/abstract]) OR (coronavirus[title/abstract]) OR (SARS[title/abstract])) AND ((pancreatitis[title/abstract]) OR (pancreas[title/abstract]) OR (pancreatic[title/abstract]) OR (lipase[title/abstract]) OR (hyperlipasemia[title/abstract]) OR (amylase[title/abstract]) OR (hyperamylasemia[title/abstract]))*. Duplicate records retrieved by both strategies were identified and removed before screening.

Two investigators independently reviewed the abstracts of all retrieved articles, excluding those that did not address COVID‐19–induced pancreatitis or lacked clinical case descriptions. The remaining studies underwent full‐text assessment. Articles without patient‐level laboratory data or not fulfilling the revised Atlanta criteria for AP [21] were excluded. For analyses focusing on “COVID‐19–induced AP,” cases were excluded if evaluation for biliary disease, alcohol‐related pancreatitis, hypertriglyceridemia, or iatrogenic causes was incomplete or not reported.

### 2.2 Data Extraction

A standardized data collection form was used to extract patient‐level information from the included studies. Variables collected included age, sex, comorbidities, symptom onset, laboratory results, imaging findings, clinical course, treatments, and survival outcomes. “Gastrointestinal symptoms” were defined according to the revised Atlanta criteria [21], as abdominal pain consistent with AP—typically persistent, moderate to severe, epigastric, deep, and sometimes radiating to the back. “Respiratory symptoms” encompassed cough, sore throat, rhinorrhea, and dyspnea. The temporal sequence of symptom onset was determined by literature review: concurrent onset was defined as gastrointestinal and respiratory symptoms reported on the same day, whereas successive onset indicated that one symptom group preceded the other by at least 1 day. When necessary, corresponding authors were contacted to clarify uncertain or ambiguous information. Overall survival was assessed from symptom onset or diagnosis of COVID‐19/AP to all‐cause mortality or last follow‐up. The study was approved by the Chang Gung Medical Foundation Institutional Review Board (IRB No. 202400636B1). The IRB reviewed the research protocol and determined that the study was exempt from further review, as it utilized information that is legally accessible and already publicly available.

### 2.3 Statistical Analyses

Categorical variables were presented as counts with percentages, and continuous variables as medians with interquartile ranges (IQR, Q3–Q1) or ranges. Patients with missing data were excluded from percentage calculations, and the corresponding denominators were reported. Statistical analyses were performed using SPSS version 26 (IBM Corp., Armonk, NY, USA). All statistical tests were two‐tailed. A *p* value < 0.05 was considered statistically significant. Pearson's χ^2^ test or Fisher's exact test was applied to categorical variables, and the Mann–Whitney U test was used for nonparametric continuous variables. Diagnostic performance was evaluated by calculating the area under the receiver operating characteristic (ROC) curve (AUC). Survival outcomes were assessed using the Kaplan–Meier method with log‐rank testing. Associations between covariates and survival were examined using univariate (unadjusted) and multivariate (adjusted) Cox proportional hazards models, with results reported as hazard ratios (HRs) and 95% confidence intervals (CIs).

## 3 Results

### 3.1 Study Population

A total of 133 articles were retrieved through the MeSH search and 691 through the title/abstract search (Figure 1). After removing 131 duplicates, 523 articles were excluded for not specifically addressing COVID‐19–associated pancreatitis or for lacking adequate exclusion of alternative AP etiologies. We further excluded 37 review articles, 18 correspondence letters without clinical cases, 12 studies without patient‐level laboratory data, 5 studies that did not meet the revised Atlanta criteria for AP, and 11 studies failing to meet both requirements. Ultimately, 87 articles comprising 111 patients were included in the analysis.

### 3.2 Patient Characteristics

The study population comprised 55 males (49.5%) and 56 females (50.5%), with a median age of 39 years (IQR, 34; range, 4–87). During hospitalization, 36 patients (32.4%) required intensive care unit (ICU) admission, and 25 (22.5%) underwent endotracheal intubation with mechanical ventilation (ETT + MV). Seven patients (6.3%) underwent surgery, while the remainder received conservative treatment. The median follow‐up from symptom onset to the end of reported documentation was 13 days (IQR, 26; range, 2–205), and from the diagnosis of either AP or COVID‐19 was 10 days (IQR, 22; range, 1–204). A total of 11 deaths were recorded. Among these, the median duration from symptom onset to death was 7 days (IQR, 7; range, 2–22), and from diagnosis of AP or COVID‐19 to death was 4 days (IQR, 3; range, 1–22).

### 3.3 Symptoms

Gastrointestinal symptoms consistent with AP were reported in 102 patients (91.9%), while respiratory symptoms were documented in 88 patients (79.3%). Among them, 34 patients (30.6%) experienced gastrointestinal symptoms before admission, whereas 58 patients (52.3%) presented with preceding respiratory symptoms. In three patients (2.7%), both symptom groups occurred concurrently, and in 16 patients (14.4%), the temporal sequence of symptom onset could not be determined from the available literature.

Among patients who developed gastrointestinal symptoms first, the median interval between gastrointestinal and respiratory symptom onset was 5 days (IQR, 7; range, 2–20), and 21 patients (61.8%) did not develop any respiratory symptoms. In contrast, among those with preceding respiratory symptoms, the median interval to gastrointestinal symptom onset was 9 days (IQR, 9; range, 1–30), with only two patients remaining free of gastrointestinal manifestations.

Among the 102 patients with gastrointestinal manifestations, 81 (79.4%) presented with typical AP symptoms before emergency department (ED) admission, whereas the remainder developed symptoms during hospitalization for COVID‐19. Table 1 compares baseline characteristics and clinical courses between these two groups. Patients who developed AP symptoms before admission were significantly younger (mean, 36 vs. 50 years; *p* = 0.020), with lower absolute lymphocyte counts (mean, 400/µL vs. 900/µL; *p* = 0.011), lower neutrophil‐to‐lymphocyte ratios (mean, 4.9 vs. 13.4; *p* = 0.029), and a higher prevalence of severe radiologic findings (Balthazar score ≥D: 56.9% vs. 27.3%; *p* = 0.027).

**Table 1:** Comparative analysis of patient characteristics and clinical trajectories stratified by pancreatitis symptom onset before admission versus during hospitalization.

Parameter	Case number	Onset of pancreatitis symptoms		*p* value
		Before admission (*n* = 81)	During hospitalization (*n* = 21)	
Age (year)	111	36 (16, 53)	50 (32, 63)	0.020*
Amylase/UNL fold	32	5.4 (2.3, 11.2)	4.6 (1.7, 6.8)	0.300
Lipase/UNL fold	48	9.6 (4.6, 18.6)	9.6 (5.4, 27.8)	0.479
Neutrophil (k/µL)	25	6.8 (3.7, 15.4)	9.4 (4.9, 23.4)	0.583
Lymphocyte (k/µL)	33	0.4 (1.0, 2.0)	0.9 (0.8, 1.0)	0.011*
N/L ratio	23	4.9 (2.3, 8.6)	13.4 (6.2, 24.5)	0.029*
Hemoglobin (g/dL)	42	13.0 (10.7, 14.5)	10.9 (9.9, 12.9)	0.098
CRP (mg/L)	69	80 (29, 172)	74 (25, 167)	0.879
AST (U/L)	50	47 (30, 102)	112 (39, 2205)	0.153
ALT (U/L)	50	48 (27, 99)	75 (40, 142)	0.231
Total bilirubin (mg/dL)	31	0.7 (0.4, 1.0)	1.7 (0.9, 3.5)	0.051
Balthazar score ≥ grade D (%)	94	41 (56.9)	6 (27.3)	0.027*
Surgical intervention (%)	110	5 (5.8)	2 (8.3)	0.645
Mortality (%)	110	7 (8.1)	4 (16.7)	0.251

*Note:* Presented by median (Q1, Q3) or number (percentage).

Abbreviations: ALT, Alanine aminotransferase; AST, Aspartate aminotransferase; CRP, C‐reaction protein; N/L ratio, neutrophil‐to‐lymphocyte ratio; UNL, upper normal limit.

* A *p* value < 0.05 was considered statistically significant.

### 3.4 Laboratory Analysis

Among all enrolled patients, complete laboratory data—including amylase and lipase levels with reported upper normal limits (UNL), as well as white blood cell (WBC) counts—were available for 22 individuals. Missing data were due to unreported values for the UNL of amylase (*n* = 38), UNL of lipase (*n* = 29), and WBC count (*n* = 46). Patients were stratified into a high WBC group (≥ 10,000/µL; *n* = 16) and a low WBC group (< 10,000/µL; *n* = 6). The amylase‐to‐UNL ratio was significantly higher in the high WBC group compared with the low WBC group (*p* = 0.003; **Appendix 1 A**). In contrast, the lipase‐to‐UNL ratio showed no statistically significant difference between groups (*p* = 0.059).

In addition, 29 patients (14 males and 15 females) had simultaneous reports of total bilirubin and alanine aminotransferase (ALT) levels. Missing data were due to unreported values for total bilirubin (*n* = 80) and ALT (*n* = 61). Female patients demonstrated a significantly higher bilirubin‐to‐ALT ratio compared with male patients (*p* = 0.007; **Appendix 1B**).

### 3.5 Imaging Survey

**Figure 2:**
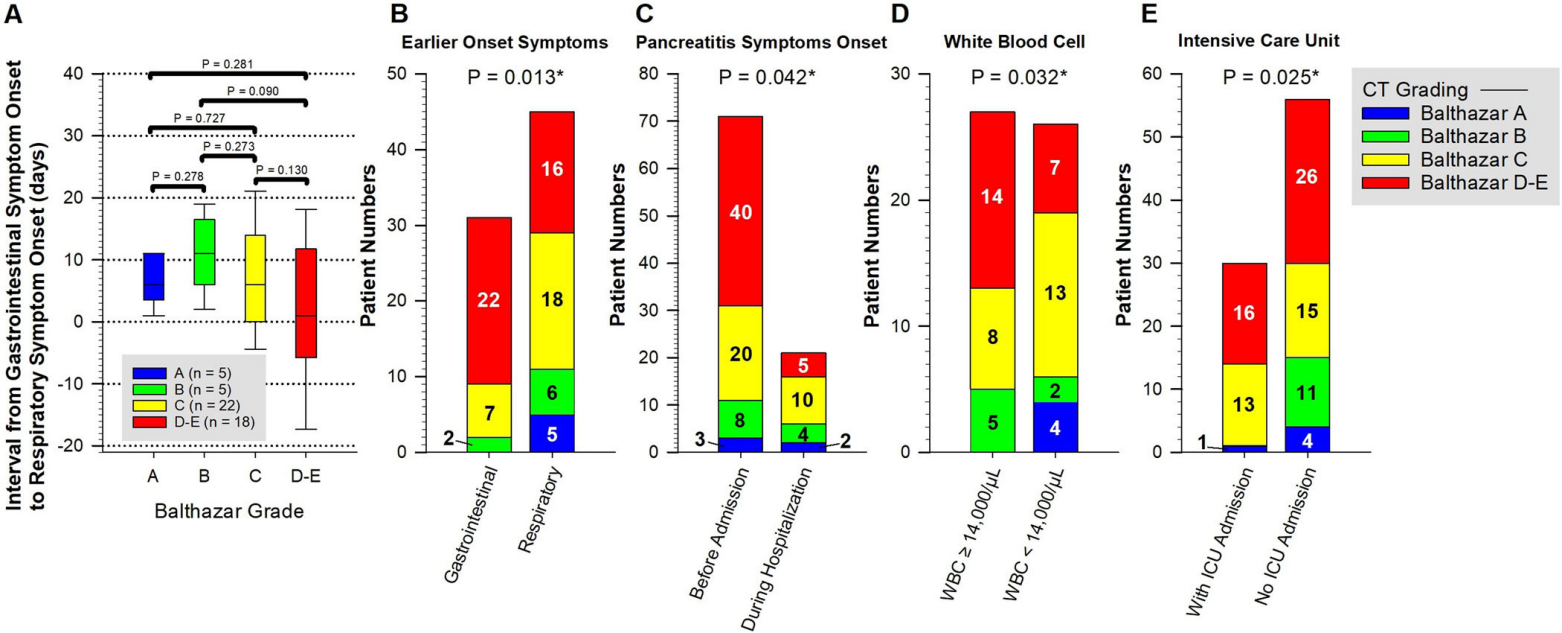
Association of Balthazar scores with the interval between gastrointestinal and respiratory symptom onset, and related clinical characteristics. (A) intergroup comparisons; (B) earlier onset symptoms; (C) pancreatitis symptoms onset; (D) white blood cell; (E) intensive care unit.

Among the 111 enrolled patients, 95 (85.6%) had available contrast‐enhanced computed tomography and/or magnetic resonance imaging reports, with characteristic findings of AP observed in 91 of 95 patients (95.8%). Based on Balthazar scoring, no significant difference in the interval between gastrointestinal and respiratory symptom onset was noted across different severity groups (Figure 2A). However, higher Balthazar scores were significantly associated with specific clinical features, including gastrointestinal symptoms preceding respiratory symptoms (*p* = 0.013), AP symptom onset before admission (*p* = 0.042), initial WBC ≥ 14,000/µL (*p* = 0.032), and ICU admission (*p* = 0.025) (Figure 2B–E).

### 3.6 Surgical Intervention

Surgical intervention was performed in seven patients (6.3%) to manage complications of AP. Among these, three underwent emergent surgery for abdominal compartment syndrome, three required debridement of intra‐abdominal organ or tissue necrosis, and one underwent emergency exploratory laparotomy for necro‐hemorrhagic pancreatitis. Despite intervention, three patients died within 2, 6, and 7 days of symptom onset, respectively.

As shown in Table 2A, ROC analysis was performed to evaluate parameters for their ability to predict the need for surgical intervention. An initial WBC ≥ 14,000/µL demonstrated excellent discriminatory performance (AUC, 0.814; 95% CI, 0.690–0.938; *p* = 0.021), while imaging‐confirmed pancreatic necrosis showed acceptable discrimination (AUC, 0.746; 95% CI, 0.519–0.973; *p* = 0.045).

**Table 2:** Receiver operating characteristic curve analyses evaluating the predictive performance for surgical intervention (Panel A) and mortality (Panel B).

Parameter	AUC (95% CI)	*p*‐value	Case number
Panel A: Requirement for surgical intervention			
Initial WBC count (k/µL)	0.753 (0.605–0.900)	0.062	64
Initial WBC ≥ 14k/µL	0.814 (0.690–0.938)	0.021*	64
Initial albumin (g/dL)	0.780 (0.536–1.000)	0.116	31
Hypocalcemia	0.795 (0.468–1.000)	0.092	42
Balthazar score ≥ grade D	0.690 (0.511–0.869)	0.091	93
Pancreatic necrosis	0.746 (0.519–0.973)	0.045*	92
Panel B: Mortality			
Age (year)	0.502 (0.335–0.669)	0.984	111
Sex	0.621 (0.454–0.789)	0.188	111
AST (U/L)	0.820 (0.611–1.000)	0.020*	50
AST ≥ 115 (U/L)	0.800 (0.585–1.000)	0.029*	50
ALT (U/L)	0.738 (0.554–0.922)	0.084	50
AST/ALT ratio	0.788 (0.578–0.999)	0.037*	47
AST/ALT ratio ≥ 2	0.752 (0.484–1.000)	0.067	47
WBC count (k/µL)	0.604 (0.237–0.972)	0.488	64
CRP (mg/L)	0.761 (0.587–0.935)	0.053	69
CRP ≥ 80 (mg/L)	0.773 (0.633–0.914)	0.043*	69
Hemoglobin (g/dL)	0.917 (0.830–1.000)	0.049*	41
Hemoglobin < 9.2 (g/dL)	0.949 (0.874–1.000)	0.034*	41
Platelet count (k/µL)	0.727 (0.573–0.882)	0.286	35
Amylase ≥ 3x UNL	0.555 (0.284–0.826)	0.685	67
Amylase/UNL ratio	0.533 (0.000–1.000)	0.876	32
Lipase ≥ 3x UNL	0.622 (0.370–0.873)	0.303	54
Lipase/UNL ratio	0.535 (0.270–0.800)	0.800	48
Albumin (g/dL)	0.833 (0.637–1.000)	0.061	31
Corrected calcium (mg/dL)	0.929 (0.794–1.000)	0.165	15
Triglyceride (mg/dL)	0.750 (0.589–0.911)	0.238	41
Balthazar score ≥ grade C	0.607 (0.442–0.772)	0.293	93
ICU admittance	0.867 (0.796–0.938)	< 0.001*	105
ETT + MV	0.929 (0.881–0.977)	< 0.001*	110
Management (conservative or surgical)	0.616 (0.418–0.814)	0.208	110

Abbreviations: ALT, Alanine aminotransferase; AST, Aspartate aminotransferase; AST/ALT ratio, AST‐to‐ALT ratio; AUC, area under the receiver operating characteristic curve; CI, confidence interval; CRP, C‐reaction protein; ETT + MV, endotracheal intubation with mechanical ventilation; ICU, intensive care unit; N/L ratio, neutrophil‐to‐lymphocyte ratio; UNL, upper normal limit; WBC, white blood cell.

* A *p* value < 0.05 was considered statistically significant.

### 3.7 Survival Outcomes

With respect to survival outcomes (Table 2B), hemoglobin (Hb) levels (AUC, 0.917, *p* = 0.049), Hb < 9.2 g/dL (AUC, 0.949, *p* = 0.034), and the requirement for ETT + MV (AUC, 0.929, *p* < 0.001) demonstrated outstanding discriminatory ability. Aspartate aminotransferase (AST) levels (AUC, 0.820, *p* = 0.020), AST ≥ 115 U/L (AUC, 0.800, *p* = 0.029), and ICU admission (AUC, 0.867, *p* < 0.001) exhibited excellent discriminatory performance. In addition, the AST‐to‐ALT ratio (AUC, 0.788, *p* = 0.037) and C‐reactive protein (CRP) ≥ 80 mg/L (AUC, 0.773, *p* = 0.043) demonstrated acceptable discriminatory capacity.

**Figure 3:**
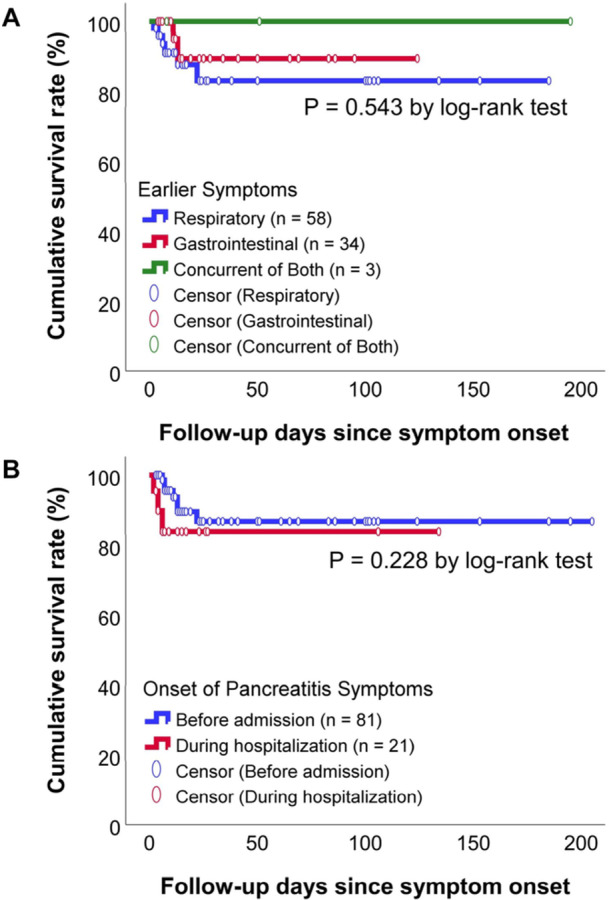
Kaplan–Meier analysis of survival outcomes in relation to symptom onset. (A) Earlier symptoms: respiratory, gastrointestinal, or concurrent of both; (B) Onset of pancreatitissymptoms: before admission or during hospitalization.

Kaplan–Meier survival analysis with the log‐rank test (Figure 3) demonstrated no significant differences in survival between patients with concurrent versus successive gastrointestinal and respiratory symptom onset (*p* = 0.543). Likewise, the timing of pancreatitis symptom onset, whether before or after hospital admission, did not significantly affect survival (*p* = 0.228).

Mortality was further evaluated using univariate and multivariate Cox proportional hazards models (Table 3). In univariate analysis, WBC count, AST‐to‐ALT ratio, AST‐to‐ALT ratio ≥ 2, hypoalbuminemia, and surgical intervention were significantly associated with mortality. After adjustment for age and sex, multivariate analysis demonstrated that WBC count (HR, 1.013; 95% CI, 1.000–1.025; *p* = 0.042), AST‐to‐ALT ratio (HR, 5.751; 95% CI, 1.291–25.624; *p* = 0.022), AST‐to‐ALT ratio ≥ 2 (HR, 11.052; 95% CI, 1.441–84.770; *p* = 0.021), and surgical intervention (HR, 6.604; 95% CI, 1.581–27.593; *p* = 0.010) remained independently correlated with survival.

**Table 3:** Univariate and multivariate Cox proportional hazards regression analyses evaluating mortality risk.

Covariate	Univariate		Multivariate (Adjusted by age & gender)	
	Unadjusted HR (95% CI)	*p* value	Adjusted HR (95% CI)	*p* value
Age (year)	0.998 (0.968–1.028)	0.874	Not performed	
Sex	0.409 (0.106–1.587)	0.196	Not performed	
Temporal sequence of symptom onset (respiratory vs. gastrointestinal)	0.436 (0.096–1.985)	0.283	Not performed	
WBC (k)	1.014 (1.003–1.026)	0.015*	1.013 (1.000–1.025)	0.042*
Hb	0.468 (0.181–1.207)	0.116	Not performed	
CRP	1.006 (0.998–1.014)	0.126	Not performed	
AST/ALT ratio	3.631 (1.079–12.222)	0.037*	5.751 (1.291–25.624)	0.022*
AST/ALT ratio ≥ 2	7.928 (1.316–47.743)	0.024*	11.052 (1.441–84.770)	0.021*
Hypoalbuminemia	3.721 (1.122–12.337)	0.032*	4.071 (0.919–18.035)	0.065
Surgical intervention	6.301 (1.626–24.428)	0.008*	6.604 (1.581–27.593)	0.010*

Abbreviations: ALT, Alanine aminotransferase; AST, Aspartate aminotransferase; AST/ALT ratio, AST‐to‐ALT ratio; CI, confidence interval; CRP, C‐reaction protein; HR, hazard ratio; WBC, white blood cell.

* A *p* value < 0.05 was considered statistically significant.

## 4 Discussion

In this study, we comprehensively analyzed the clinical courses and outcomes of 111 patients with COVID‐19–induced AP. Pancreatitis symptom onset before hospital admission was significantly associated with younger age, lower absolute lymphocyte counts, reduced neutrophil‐to‐lymphocyte ratios, and a higher likelihood of severe radiologic findings (Balthazar score ≥D). Patients with elevated WBC counts (≥ 10,000/µL) exhibited significantly higher amylase‐to‐UNL ratios, whereas lipase‐to‐UNL ratios did not differ. The interval between gastrointestinal and respiratory symptom onset was not correlated with imaging severity; however, higher Balthazar scores were observed in patients with gastrointestinal symptoms preceding respiratory symptoms, AP symptom onset before admission, initial WBC ≥ 14,000/µL, and ICU admission. Initial WBC ≥ 14,000/µL and pancreatic necrosis were predictive of the need for surgical intervention, while survival outcomes were associated with Hb, AST, CRP, requirement for ETT + MV, and the AST‐to‐ALT ratio. In multivariate Cox regression models adjusted for age and sex, WBC count, AST‐to‐ALT ratio, AST‐to‐ALT ratio ≥ 2, and surgical intervention remained independently correlated with mortality.

Focusing on patients with COVID‐19–induced AP, this study comprehensively examined the relationships between symptom onset and other clinical variables. AP diagnosis was primarily based on the presence of characteristic clinical symptoms. During the global COVID‐19 pandemic, the interval from disease onset to confirmed diagnosis varied widely for both AP and COVID‐19. Differences in healthcare accessibility and national quarantine policies inevitably introduced bias when using documented diagnosis time to estimate “true disease onset.” Therefore, reported symptom onset serves as a valuable proxy for assessing the temporal course of illness.

Notably, although Kumar et al. [22] observed a similar proportion of patients with early respiratory symptoms (52.9%), all developed SARS‐CoV‐2–associated respiratory distress syndrome requiring mechanical ventilation, whereas only 9 of 58 patients (15.5%) in our cohort required ETT + MV. Differences in baseline characteristics were also apparent: Kumar et al.'s patients were younger (median 57 vs. 63 years), predominantly female (55.6% vs. 25%), Hispanic (55.6% vs. 25%), and obese (88.9% vs. 37.5%), whereas in our study, patients admitted for respiratory symptoms were significantly older (median 50 vs. 36 years). Overall, patients presenting first with respiratory symptoms showed comparable ages across studies, whereas those with early gastrointestinal symptoms exhibited marked differences. Determining whether the severity of respiratory symptoms influences the interval to gastrointestinal manifestations remains challenging, and observed discrepancies may reflect patient heterogeneity, variable SARS‐CoV‐2 variants, and incomplete reporting of disease phenotypes.

In our review‐based synthesis cohort, 52.3% of patients reported respiratory symptoms preceding gastrointestinal manifestations, with a median interval of 9 days (range, 1–30). Kumar et al. [22] reported a median duration of 22.5 days (range, 13–76) from COVID‐19 symptom onset to the development of AP. A review encompassing 37 patients described a bimodal pattern: 43% first presented with respiratory symptoms, 43% with AP symptoms, and 14% with concurrent onset [23], supporting a dual pathogenesis involving cytotoxic and immune‐mediated pancreatic injury. Notably, although Kumar et al. observed a similar proportion of patients with early respiratory symptoms (52.9%), all developed SARS‐CoV‐2–associated respiratory distress syndrome requiring mechanical ventilation, whereas only 9 of 58 patients (15.5%) in our cohort required ETT + MV. Differences in baseline characteristics were also apparent: Kumar et al.'s patients were younger (median 57 vs. 63 years), predominantly female (55.6% vs. 25%), Hispanic (55.6% vs. 25%), and obese (88.9% vs. 37.5%), whereas in our study, patients admitted for respiratory symptoms were significantly older (median 50 vs. 36 years). Overall, patients presenting first with respiratory symptoms showed comparable ages across studies, whereas those with early gastrointestinal symptoms exhibited marked differences. Determining whether the severity of respiratory symptoms influences the interval to gastrointestinal manifestations remains challenging, and observed discrepancies may reflect patient heterogeneity, variable SARS‐CoV‐2 variants, and incomplete reporting of disease phenotypes.

Numerous studies have examined the occurrence of AP among patients with COVID‐19. Early reports suggested pancreatic injury or biochemical abnormalities in 8.5%–17% of cases [11, 12], yet subsequent investigations identified a markedly lower incidence of true AP meeting the revised Atlanta criteria [21]. In a prospective cohort of 433 patients with COVID‐19, only five cases (1.2%) of AP were documented [24]. Interestingly, the proportion of idiopathic AP appears to be higher in the presence of COVID‐19 [19, 20]. Although consensus on the diagnosis of COVID‐19–induced AP is lacking, rigorous exclusion of alternative etiologies remains essential. Further studies are warranted to elucidate the potential contribution of coinfections and other pathogens, which may independently trigger AP through diverse mechanisms in this setting.

With respect to AP severity and outcomes, two retrospective studies evaluated 189 [25] and 54 [26] patients presenting to the emergency department with AP. In both cohorts, concurrent COVID‐19 was associated with increased AP severity and mortality. In patients with comorbid COVID‐19, Dirweesh et al. [18] reported higher Charlson comorbidity indices, elevated BISAP scores, multiorgan failure, and consequently increased mortality. Inamdar et al. [20] observed prolonged hospitalization and a higher requirement for mechanical ventilation. These findings were further supported by a prospective multicenter study [27], which confirmed that patients with both AP and COVID‐19 experienced higher mortality, greater organ failure rates, and increased in‐hospital death compared with those without COVID‐19. Collectively, the coexistence of AP and COVID‐19 may contribute to adverse clinical outcomes. Importantly, systemic effects induced by COVID‐19, such as respiratory injury, could independently jeopardize patient survival. Whether these observations reflect the severity of true “COVID‐19–induced AP” remains uncertain. Clear and globally standardized definitions distinguishing “COVID‐19–induced AP” from “concurrent COVID‐19 and AP” are critical, as the latter encompasses a heterogeneous population including AP secondary to biliary disorders, alcohol, hypertriglyceridemia, or iatrogenic causes. By enrolling only cases with rigorous exclusion of alternative AP etiologies, the present study provides insight into the authentic clinical course of COVID‐19–induced AP. Standardized terminology is essential for future accumulation and comparison of global clinical experiences, minimizing bias and enhancing interpretability.

Several limitations of this study merit consideration. The retrospective extraction of data from published reports introduces inherent limitations, including potential selection bias. Reliance on patient‐reported symptom onset and other clinical details may increase recall bias. Additionally, second‐hand reporting bias cannot be entirely excluded, although efforts were made to clarify uncertain or ambiguous information through correspondence with original authors. Despite these measures, unavoidable factors—including differences in SARS‐CoV‐2 variants, temporal and geographic variability, vaccination coverage, healthcare accessibility, and regional quarantine policies—contributed to heterogeneity across the study population, potentially affecting hospital stay duration and specific care practices. To mitigate these issues, our analyses focused on objectively reported variables, such as documented symptoms, laboratory results, and imaging findings. Furthermore, a comprehensive assessment of AP etiology was constrained by the limitations of the published literature. Although most reports excluded common etiologies and provided clinical information, in some cases, authors attributed AP to COVID‐19–associated systemic inflammation after ruling out conventional causes, without detailing the diagnostic procedures employed. As different etiologies of AP can result in distinct clinical presentations [28], this introduces an additional layer of heterogeneity. Finally, follow‐up was restricted to the period reported in the literature, limiting the evaluation of long‐term outcomes.

## 5 Conclusion

COVID‐19–induced AP is associated with substantial short‐term mortality. In contrast to “concurrent COVID‐19 and AP” without exclusion of established etiologies, the timing of gastrointestinal or respiratory symptom onset—before or after hospitalization—did not significantly influence survival in patients with COVID‐19–induced AP. Comprehensive analysis of clinical parameters, including symptoms, laboratory and imaging findings, and surgical interventions, identified leukocytosis, elevated AST‐to‐ALT ratio, and surgical intervention as significant predictors of mortality, providing novel insights from real‐world data. These findings underscore the critical need for standardized terminology distinguishing “COVID‐19–induced AP” from “concurrent COVID‐19 and AP,” as the two entities represent distinct pathophysiological and clinical constructs. Clear definitions are essential for enabling accurate comparisons across global clinical experiences and minimizing interpretive bias in future research.

## Author Contributions

Conceptualization: Yu‐Jhou Chen, Tsung‐Hsing Chen, Chen‐June Seak, Chang‐Mu Sung, Hao‐Tsai Cheng; Methodology: Yu‐Jhou Chen, Tsung‐Hsing Chen, Hao‐Tsai Cheng; Funding acquisition: Hao‐Tsai Cheng; Data curation: All authors; Validation: Yu‐Jhou Chen, Tsung‐Hsing Chen, Chen‐June Seak, Hao‐Tsai Cheng; Formal analysis and investigation: All authors; Visualization: Yu‐Jhou Chen, Hao‐Tsai Cheng; Writing – original draft: Yu‐Jhou Chen, Hao‐Tsai Cheng; Writing – review and editing: All authors; Supervision: Chen‐June Seak, Chang‐Mu Sung, Shu‐Wei Huang, Sen‐Yung Hsieh.

## Disclosure

The first author, Yu‐Jhou Chen, affirms that this manuscript is an honest, accurate, and transparent account of the study being reported; that no important aspects of the study have been omitted; and that any discrepancies from the study as planned (and, if relevant, registered) have been explained.

## Consent

The patient‐level data utilized in this review‐based synthesis were extracted from previously published literature.

## Conflicts of Interest

The authors declare no conflicts of interest.

## Data Availability

All authors had full access to all of the data in this study and takes complete responsibility for the integrity of the data and the accuracy of the data analysis. The data that support the findings of this study are available on request from the corresponding author. The data are not publicly available due to privacy or ethical restrictions.
